# Cohort profile: the SEROCoV-KIDS population-based cohort study and biobank on the impact of the COVID-19 pandemic in children and adolescents in Geneva, Switzerland

**DOI:** 10.1136/bmjopen-2026-116875

**Published:** 2026-09-21

**Authors:** Elsa Lorthe, Andrea Loizeau, Viviane Richard, Roxane Dumont, María-Eugenia Zaballa, Francesco Pennacchio, Julien Lamour, Arnaud G L’Huillier, Hélène Baysson, Natalia Fernandez Clares, Nicolas Bovio, Mayssam Nehme, Pierre Lescuyer, Nicolas Vuilleumier, Klara M Posfay-Barbe, Rémy P Barbe, SEROCoV-KIDS study group, Deborah Amrein, Idris Guessous, Silvia Stringhini

**Affiliations:** 1 Department of Primary Care Medicine, Geneva University Hospital, Geneva, Switzerland; 2 Université Paris Cité, Inserm, Inrae, Obstetric, Perinatal, Paediatric Life Course Epidemiology (OPPaLE) Research Team, Center for Research in Epidemiology and Statistics (CRESS), Paris, Île-de-France, France; 3 Geneva School of Health Sciences, HES-SO University of Applied Sciences and Arts Western Switzerland, HES-SO Geneve, Geneva, Switzerland; 4 Department of Women, Child and Adolescent Medicine, Pediatric Infectious Diseases Unit, Geneva University Hospitals and Faculty of Medicine, Geneva, Switzerland; 5 Department of Diagnostics, Laboratory of Virology, Geneva University Hospitals, Geneva, Switzerland; 6 Department of Pediatrics, Gynecology and Obstetrics, Faculty of Medicine, University of Geneva, Geneva, Switzerland; 7 Department of Health and Community Medicine, Faculty of Medicine, University of Geneva, Geneva, Switzerland; 8 Division of Laboratory Medicine, Geneva University Hospitals, Geneva, Switzerland; 9 Department of Medicine, Faculty of Medicine, University of Geneva, Geneva, Switzerland; 10 Division of General Pediatrics, Department of Woman, Child, and Adolescent Medicine, Geneva University Hospitals, Geneva, Switzerland; 11 Division of Child and Adolescent Psychiatry, Department of Woman, Child, and Adolescent Medicine, Geneva University Hospitals, Geneva, Switzerland; 12 School of Population and Public Health and Edwin S.H. Leong Centre for Healthy Aging, Faculty of Medicine, University of British Columbia, Vancouver, British Columbia, Canada

**Keywords:** COVID-19, Child, Adolescent, Health

## Abstract

**Abstract:**

**Purpose:**

The COVID-19 pandemic raised major concerns about its potential effects on children and adolescents, particularly through disruptions to their health, social and educational environments and the exacerbation of existing inequalities. In response to the limited evidence-based knowledge available at the time, the SEROCoV-KIDS study was launched in 2021 as a prospective cohort and biobank to assess the pandemic’s effects on youth health and well-being. It focuses on the general population of Geneva, Switzerland, as well as subgroups with vulnerabilities, including prior SARS-CoV-2 infection, chronic health conditions and socioeconomic disadvantage.

**Participants:**

A total of 2199 children and adolescents, aged 6 months to 17 years, from 1340 households were enrolled in the SEROCoV-KIDS cohort, 2048 from the general population and 151 from clinics (ie, children with chronic health conditions). At baseline, between December 2021 and June 2022, participants provided blood samples for serological testing and for long-term storage in the biobank, while comprehensive questionnaires were completed by a referent adult and adolescents aged 14 and older. Five follow-up online assessments were conducted until October 2026, addressing physical and mental health, development, quality of life, health behaviours, family dynamics and education-related topics. The quantitative findings of the cohort study were enriched by a cross-sectional qualitative study conducted between October 2023 and March 2024, focusing specifically on socioeconomically disadvantaged populations.

**Findings to date:**

In the population-based sample, 66.3% of participants tested seropositive for anti-SARS-CoV-2 nucleocapsid antibodies at baseline, and 4.1% reported symptoms consistent with post-COVID-19 condition. Most children were minimally or not at all affected by the pandemic, showing good mental health over time. However, 8% of participants reported a positive pandemic impact, whereas 8%–13% experienced negative impacts, mainly due to disrupted routines and reduced social support. Health behaviours like physical activity and sleep remained largely stable over the study period. Higher screen time at baseline was associated with poorer well-being. Children with chronic health conditions or experiencing socioeconomic and family disadvantage were disproportionately affected in terms of the health and psychosocial consequences of the pandemic.

**Future plans:**

SEROCoV-KIDS demonstrates the value of child-focused cohorts for understanding the consequences of major societal events and for guiding evidence-based policy. Our next priority is to secure funding to prolong follow-up of this cohort and to maintain systematic surveillance of children and adolescents, so that emerging findings can directly inform public health and education policy over the coming years.

**STRENGTHS AND LIMITATIONS OF THIS STUDY**

- This large population-based paediatric cohort provides insights into the health and well-being of children and adolescents aged 6 months to 17 years at baseline, during and after the COVID-19 pandemic (from December 2021 to October 2026).
- This study integrates multifaceted findings on the general paediatric population and on subgroups of children with clinical and/or social vulnerabilities, combining quantitative and qualitative data to provide a deeper understanding of how the pandemic has affected their lives.
- This unique paediatric cohort and its associated biobank offer a rare opportunity to advance future paediatric research in Switzerland and abroad.
- Generalisability may be limited, as participating families tended to be more highly educated and somewhat more socioeconomically advantaged than the general Geneva population, despite nearly one-fifth reporting financial difficulties.
- The study was designed in response to the pandemic, and individual-level pre-pandemic data are lacking, which limits direct comparisons over time, relying instead on parent-reported perception of changes and impacts.

## Introduction

 The COVID-19 pandemic has had far-reaching and multifaceted effects on global populations, with children and adolescents experiencing unique and evolving challenges across health, social and educational domains. During the early stages of the pandemic, marked by high mortality rates and a sense of emergency, research primarily focused on understanding the pathophysiology of the disease in its acute phase, its transmission, diagnosis, treatment and prevention. Preliminary evidence showed that children and adolescents had lower susceptibility to SARS-CoV-2 infection compared with adults, and played a lesser role than adults in transmission of SARS-CoV-2 at a population level.^1^ Little attention was given to the paediatric population, as children represented only a small fraction of diagnosed SARS-CoV-2 infections,^2 3^ and an even smaller fraction of severe cases.^3–5^ This was potentially linked to changes in contact patterns related to school closures, and underdiagnosis due to the scarcity of tests available, as well as the high frequency of asymptomatic infections or non-specific mild symptoms in children.^1^ However, with the emergence of variants of concern, it became evident that children could be infected and reinfected, and could transmit the infection to their households and classmates.^6–13^ Severe complications, such as multisystem inflammatory syndrome and post-COVID-19 condition, were also increasingly reported, with a disproportionately higher burden observed among children with pre-existing health conditions.^3 14–16^

While early research primarily focused on children’s susceptibility to infection and risk of severe disease, attention increasingly shifted towards the broader consequences of public health measures and societal disruptions on child and adolescent development and well-being. As the crisis unfolded, it became clear that its impact extended far beyond the direct effects of infection, reshaping social, educational and family environments that are critical to healthy development. Children and adolescents were largely affected, as their lives were profoundly disrupted by the extensive measures implemented to control the spread of the pandemic. School closures, social isolation, changes in family dynamics, unhealthy behaviours such as prolonged screen time or reduced physical activity, and generalised uncertainty posed significant challenges.^17–20^ This was particularly salient for older children and adolescents, for whom peer interactions are critical to development.^17 21^ Younger children were also highly vulnerable, as they faced prolonged stress during key developmental stages and often lacked the internal and external resources to cope adequately.^22–24^ While early meta-analyses showed only minimal increases in mental health symptoms among youth,^25 26^ subsequent studies reported a growing frequency of mental health issues, especially among girls and older adolescents, likely due to repeated lockdowns and lifestyle disruptions.^22 27–32^ However, not all outcomes were negative. In some cases, lockdowns fostered stronger family bonds and offered opportunities for more individualised learning experiences.^33 34^ Given these complex and evolving dynamics, it was essential to examine the pandemic’s mid- and long-term impact on the mental health and well-being of children and adolescents.

As evidence accumulated regarding these broader consequences for children and adolescents, concerns also emerged about their unequal distribution across social groups. Indeed, since the earliest months of the pandemic, it became apparent that the pandemic not only revealed but also intensified existing social inequalities, disproportionately impacting disadvantaged populations.^35–37^ Adults from low-income, immigrant or minority backgrounds were more likely to experience both direct (eg, SARS-CoV-2 infection, severe COVID-19 disease, hospitalisation and death) and indirect (eg, stress, mental health issues, job insecurity and financial difficulties) consequences of the pandemic.^35 38^ Among children and adolescents, factors such as poverty, low parental education, immigrant background or racial/ethnic minority background were identified as risk factors for poor mental health outcomes,^39^ both prior to and throughout the COVID-19 pandemic.^17 40–42^ Gaining a deeper understanding of the social patterning of the pandemic long-term effects was essential for mitigating its enduring impact on youth well-being.

In addition to its psychological and social toll, the pandemic disrupted education, with school closures and remote learning contributing to worsening academic performance^43 44^ and widening educational disparities.^45^ While some children, particularly those from higher socioeconomic backgrounds, were able to maintain or even advance their learning through parental support and digital access, many disadvantaged students faced barriers such as limited internet access, lack of learning space, reduced educational support at home and diminished access to school-based services.^46^ These disparities risk deepening pre-existing learning gaps and may have long-term consequences on academic achievement and future opportunities.

Recognising the scarcity of data available at the beginning of the pandemic and anticipating the need for robust evidence to guide public health policies, in 2021 we launched SEROCoV-KIDS as a prospective cohort study and biobank to evaluate the impact of the COVID-19 pandemic on the health and well-being of children and adolescents. Its specific objectives were: (1) to assess the prevalence of anti-SARS-CoV-2 N and S antibodies in a paediatric population-based study; (2) to investigate the pandemic’s health and psychosocial effects on children and adolescents from the general population and (3) to examine outcomes among subgroups with vulnerabilities, including prior SARS-CoV-2 infection, chronic health conditions and/or socioeconomic disadvantage. We hypothesised that children’s health and well-being were both directly and indirectly affected by the pandemic, with disproportionately greater impacts observed in subgroups with clinical and/or social vulnerabilities.

## Cohort description

### Study setting and context of the COVID-19 pandemic

The first confirmed COVID-19 case was reported in the canton of Geneva, Switzerland, on 26 February 2020.^47^ In response to the surge in cases, a partial lockdown was implemented on 16 March 2020, including school closures until 11 May 2020. Thereafter, despite experiencing multiple COVID-19 waves, Switzerland generally prioritised keeping schools and certain activities for children and adolescents open, while adjusting public health measures according to the epidemiological situation. By February 2022, most restrictions had been lifted, and all remaining measures were removed by May 2022. Vaccination became available to children aged 12 and older in June 2021 and to those aged 5 and older in January 2022.^48^

### Cohort study design, population and recruitment procedures

**Figure 1:**
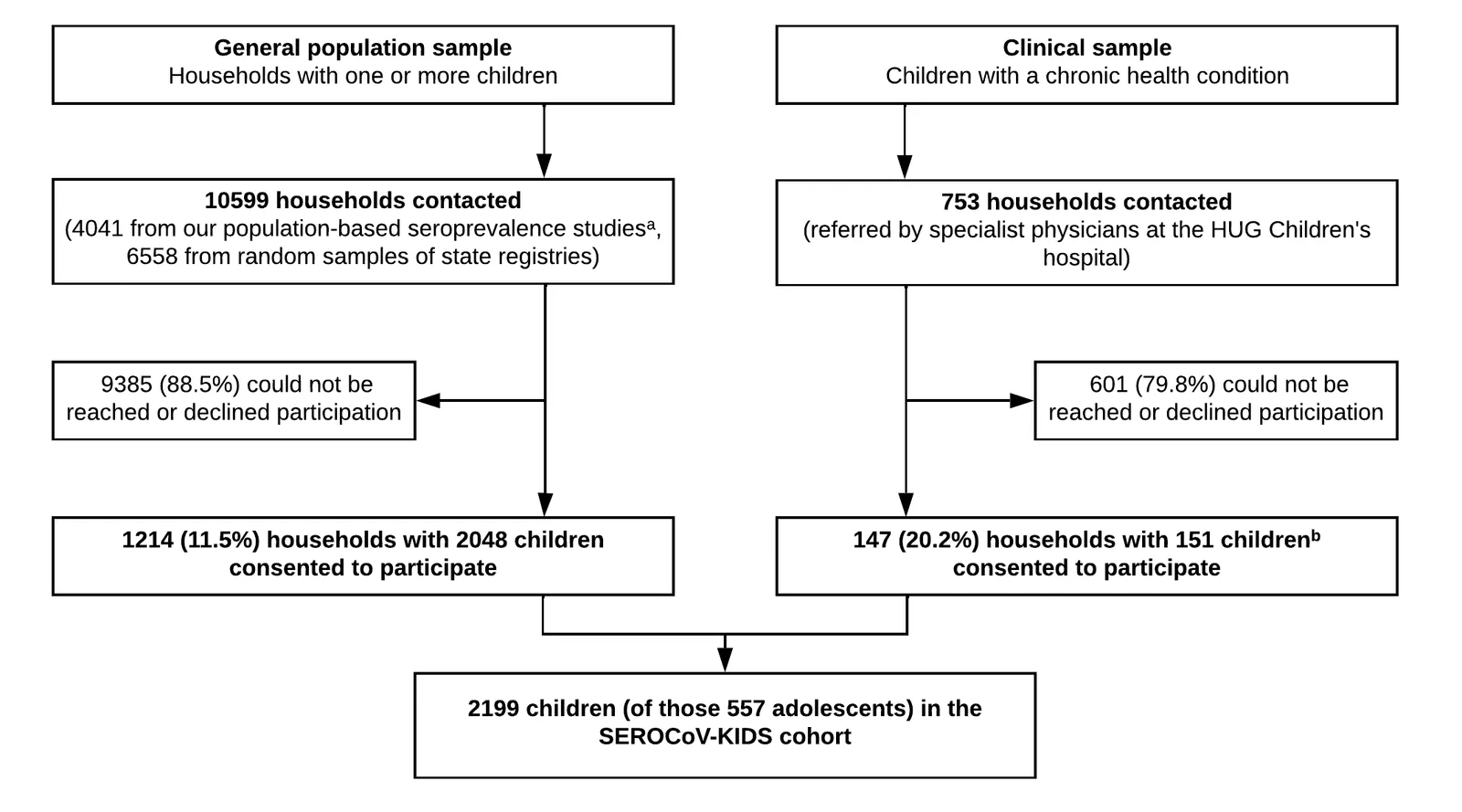
Flow chart of the study. HUG, University Hospitals of Geneva. ^a^For 1802 former participants, the presence or absence of children in the household was unknown at the time of invitation. ^b^Proportions of children enrolled per hospital unit: 10 (6.6%) immunology, 63 (41.7%) pneumonology, 31 (20.5%) allergology, 10 (6.6%) gastroenterology, 26 (17.2%) neurology and 11 (7.3%) rheumatology.

This prospective cohort study of children and adolescents includes both a general population sample and a clinical sample of children with chronic health conditions (figure 1). For the population-based sample, eligible children and adolescents were those living in the canton of Geneva and aged 6 months to 17 years at baseline. Participants were randomly selected from state registries by the Swiss Federal Office of Statistics or the Cantonal Office for Population and Migration, either specifically for this study or for other COVID-19 population-based seroprevalence studies conducted by our group.^47–50^ The selected individuals were then invited to participate by our team, along with their siblings who met the eligibility criteria. The sample size was determined to ensure adequate statistical power for comparisons between key subgroups defined by serological status, age group, socioeconomic level and the presence of chronic disease or disability. Subgroup sample sizes ranging from 100 to 1000 participants were expected to provide at least 80% power to detect differences of 5%–10% between groups, depending on the prevalence of the outcome.

Children from the clinical sample were referred by specialist physicians working at the Children’s Hospital (Geneva University Hospitals), the main paediatric referral centre in the canton of Geneva. This recruitment strategy was implemented to ensure adequate representation of children with a broad range of chronic health conditions, who might otherwise have been underrepresented in the population-based sample. Physicians from the pneumology, immunology, allergology, gastroenterology, neurology and rheumatology units invited eligible patients to participate. Children were eligible if they met the same age and residency criteria and had one or more medically diagnosed conditions lasting for at least 6 months. These included, but were not limited to, chronic respiratory or cardiac diseases, severe allergies, neurological disorders, rheumatic diseases, diabetes, inflammatory bowel disease or immune system disorders.

A pilot phase was conducted in November 2021 among 130 children and adolescents of participants in a previous seroprevalence study,^51^ allowing us to validate all study procedures. Participants enrolled in the pilot phase were not included in the main cohort, unless they were also randomly selected for the main cohort. Recruitment for the main study took place between December 2021 and June 2022 for the population-based sample and between June and December 2022 for the clinical sample.

Adolescents from 14 years of age and parents (or legal guardians) of participants provided electronic or written informed consent at baseline. Children gave oral assent to participate. Participants who reached 14 years of age were individually invited to provide consent in June 2022 and in September 2023. Among them, 30 (49.2% of those contacted) agreed to complete their own questionnaires. Without opt-out requests, all participants were followed throughout the data collection period, including those transitioning into adulthood.

### Cohort study procedures, blood collection and questionnaires

The baseline assessment included the completion of online inclusion questionnaires and an optional serological test offered to all children and adolescents. To detect anti-SARS-CoV-2 antibodies, we used two highly accurate immunoassays: the Roche Elecsys anti-SARS-CoV-2 spike (S) and anti-SARS-CoV-2 nucleocapsid (N) assays (Roche Diagnostics, Rotkreuz, Switzerland). Seropositivity was determined using the manufacturer’s cut-off values: ≥0.8 U/mL for the S assay and index ≥1.0 for the N assay. COVID-19 exposure data were collected through a brief paper survey administered during the baseline visit.

An additional blood sample was collected from each child for long-term storage in the SEROCoV-KIDS biobank. All samples were labelled with unique identification numbers and stored at the Diagnostics Department of the Geneva University Hospitals. For participants whose referent adult provided consent for extended use of biospecimens, samples were preserved in the biobank for future analyses. Biobank regulation follows the guidelines of the Swiss Biobanking Platform.

At baseline, one referent adult per family completed a detailed digital questionnaire covering socio-demographic, health, lifestyle, well-being and COVID-19 pandemic-related information for each participating child, as well as for themselves and their household. Adolescents aged 14 and older independently completed an age-appropriate questionnaire about their health and well-being. All questionnaires were completed online via the Specchio-Hub digital platform (https://www.specchio-hub.ch/),^52^ with a small number of participants opting for a paper-based format.

Annual follow-up questionnaires were administered in late spring 2023, 2024 and 2025 to monitor trends in physical and mental health, quality of life, development and lifestyle over time. Validated scales were used whenever possible (tables 1 and 2) and translated into French through a forward-backward translation approach if necessary. Additionally, thematic questionnaires were distributed in autumn 2022 and spring 2024 to explore family-related and education-related topics, respectively. At each time point, adolescents completed an age-appropriate questionnaire. All questionnaires were designed to be completed online within 15–30 min. In the case of non-response, up to three email reminders were sent, spaced 7–14 days apart, followed by a phone call and/or a paper reminder if needed. To encourage participation, families were entered into a raffle for small incentives (eg, vouchers, movie tickets) after each annual questionnaire. Retention strategies also included regularly sharing study findings via email newsletters or webinars and distributing paper trifold flyers tailored to the young population.

**Table 1:** Children data collection over the course of the SEROCoV-KIDS study

Data collection period*	Year 1	Year 2		Year 3		Year 4
	Baseline	Thematic Q I	Annual Q I	Thematic Q II	Annual Q II	Annual Q III
Serology assessment† and biobank						
Successful blood draw, visit date	x					
Presence of anti-SARS-CoV-2 (S/N antibodies)	x					
Biobank agreement	x					
Children characteristics‡						
COVID-19 exposure§						
Vaccination, infections, symptoms	x	x	x		x	
Symptom disability (Sheehan)			x		x	
General and mental health						
Parent-rated general health	x	x	x	x	x	x
Medical conditions, height, weight	x		x		x	x
Hospitalisation			x		x	x
Medication, oral health			x		x	
Strengths and difficulties (SDQ)	x		x		x	x
Specific healthcare needs (CSHCN)		x			x	
Quality of life (PedsQL)	x		x		x	x
Fatigue (PedsQL-MFS)			x		x	x
Development (ASQ-3)	x		x		x	
Education						
Educational level, learning	x			x		x
Support, school type	x			x		
Parenting (PAFAS 1,2)		x				
School functioning (PedsQL subscale, MSLSS)				x		x
Parental involvement (QIPSS), expectations				x		
Bullying, child anxiety (CAIS)				x		x
Environment						
Relationships	x		x	x	x	x
Exposure to nature	x		x			x
Social support (MSPSS), work-family conflict (ISSP)		x			x	
Life events (LEQ)			x		x	x
Lifestyle						
Physical and leisure activities, screen use	x		x	x	x	x
Screen addiction, parental screen mediation						x
Eating and sleep behaviours	x		x		x	x
Healthcare access						
Healthcare renunciation	x		x		x	
Pandemic impact						
Self-rated general impact	x	x	x	x		
Coronavirus impact scale	x					
Pandemic family impact (CEFIS)		x				
Household characteristics						
Sociodemographic characteristics¶	x			x	x	
Educational level, occupational status	x			x		
Medical history	x					

When appropriate, bilingual experts in epidemiology performed English (EN) to French (FR) translations using a forward-backward approach.

* Data are collected at baseline and every 6 months to 1 year.

† Data are collected at baseline from three sources: participant chart, laboratory analyses and consent form.

‡ Parent-reported.

§ Data were also extracted from a 5-min paper survey administered during the serological visit.

¶ Abstracted from the referent adult registration questionnaire.

ASQ-3, Ages and Stages Questionnaires (FR, age ≤5 years old); CAIS, Child Anxiety Impact Scale (EN, subscale); CEFIS, COVID-19 Exposure and Family Impact Scales (EN); CSHCN, Children with Special Health Care Needs (EN); ISSP, Work-Family Conflict Scale (FR); LEQ, Lifetime of Experiences Questionnaire (EN); MSLSS, Multidimensional Student’s Life Satisfaction Scale (EN, subscale, adapted); MSPSS, Multidimensional Scale of Perceived Social Support (FR); PAFAS 1,2, Parenting and Family Adjustment Scales (EN, age ≥2 years old); PedsQL, Paediatric Quality of Life Inventory (FR, age ≥2 years old); PedsQL-MFS, Paediatric Quality of Life Inventory-Multidimensional Fatigue Scale (FR, age ≥2 years old); Q, Questionnaire; QIPSS, Parental Involvement Questionnaire (FR, subscale); SDQ, Strengths and Difficulties Questionnaire (FR, age ≥2 years old); Sheehan, Sheehan disability scale (FR, adapted).

**Table 2:** Adolescents data collection over the course of the SEROCoV-KIDS study

Data collection period*	Year 1	Year 2		Year 3		Year 4
	Baseline	Thematic Q I	Annual Q I	Thematic Q II	Annual Q II	Annual Q III
Adolescent characteristics†						
COVID-19 exposure						
Vaccination, perception	x					
General and mental health						
Self-rated general health	x			x	x	x
Height, weight		x			x	x
Gender identity, menstrual cycle					x	
Quality of life (PedsQL), well-being (WHO-5)	x		x		x	x
Self-esteem (RSES)	x		x		x	x
Distress (IDPSQ-14), suicidal ideation	x		x		x	x
Depression (PHQ-9)					x	x
Self-perception		x	x		x	
Personality (Big5)		x				
Happiness (SHS), future prospects (FTP)		x				
Acne (GEA, AcneQ4)			x			
Resilience (BRS)			x		x	x
Education						
Educational level, learning, support	x			x		x
School well-being (BASWBSS), burn-out (SBI)				x		x
Bullying (BVQr)	x		x	x		
Environment						
(Love) relationships	x	x	x		x	x
Social support (MSPSS)	x		x		x	x
Life events (PANALE)				x		
Geopolitical awareness, eco-anxiety (HEAS)				x		
Lifestyle						
Physical activities (IPAQ)	x	x		x		x
Leisure activities, screen use	x		x	x	x	x
Artificial intelligence use			x	x		x
Gaming (IGCS)						x
Eating behaviours	x	x				x
Sleep behaviours (year 3, ISI-3)	x				x	x
Cyber-bullying, social networks (BSMAS)	x		x		x	x
Addictions	x		x		x	x
Pandemic impact						
Self-rated general impact	x			x		

When appropriate, bilingual experts in epidemiology performed English (EN) to French (FR) translations using a forward-backward approach.

* Data are collected at baseline and every 6 months to 1 year.

† Self-reported.

AcneQ4, Acne four-Question Scale (FR); BASWBSS, Brief Adolescents' Subjective Well-Being in School Scale (EN); Big5, Big five-factor personality model (FR); BRS, Brief Resilience Scale (FR); BSMAS, Bergen Social Media Addiction Scale (FR); BVQr, Olweus revised Bully and Victim Questionnaire (FR); FTP, Future Time Perspective Scale (FR); GEA, Global Evaluation Acne (FR); HEAS, Hogg Eco-Anxiety Scale (EN); IDPSQ-14, Quebechealth Department's Psychological Distress Index (FR); IGCS, Internet Gaming Cognitions Scale (FR); IPAQ, International Physical Activity Questionnaire (FR); ISI-3, Insomnia Severity Index – brief version (FR); MSPSS, Multidimensional Scale of Perceived Social Support (FR); PANALE, Positive And Negative Adolescent Life Experiences Scale (EN); PedsQL, Pediatric Quality of Life Inventory (FR); PHQ-9, Patient Health Questionnaire for Depression Severity (FR); RSES, Rosenberg Self-Esteem Scale (EN); SBI, School Burnout Inventory (FR); SHS, Subjective Happiness Scale (FR); WHO-5, WHO well-being index.

### Qualitative study design, population and methods

The quantitative findings of the cohort study were complemented and enriched by a cross-sectional qualitative study conducted between October 2023 and March 2024, focusing specifically on socioeconomically disadvantaged populations. This component, known as the PIRA project (Pandemic and Inequalities: Analysis of the Impact on Adolescents), was carried out by an external team of experts commissioned for this purpose, under a mandate assigned to the Observatory of Families, affiliated with the Institute of Sociological Research in Geneva.

Eligibility criteria included being 10–14 years old during the acute phase of the pandemic, and either reporting negative impacts from the pandemic or living in a low-income family, a household experiencing family tensions or conflicts or having a family member facing health issues or disabilities.^53^ Recruitment took place in facilities offering recreational activities and individual and group support for young people, in various neighbourhoods and municipalities of the canton of Geneva, as well as in municipal social services and consultation centres frequented by the parents of the targeted adolescents. Contact was established through a playful activity that helped initiate discussion and assess the presence of at least one vulnerability criterion, which led to proposing participation in the study. Semistructured qualitative interviews were conducted to gather first-hand accounts and gain a deeper understanding of how young people experienced the period from the initial lockdown to the lifting of all restrictions, as well as any challenges they faced up to the time of the interview.

An additional component of this qualitative study involved focus groups with frontline professionals who have been working with adolescents aged 10–18 since 2020, including teachers, specialised educators, psychologists and social workers. Frontline professionals were recruited through the research team’s professional network, using snowball sampling, and by contacting the management of relevant institutions and community organisations. These organisations were asked to identify and invite professionals who met the study eligibility criteria. Participants were invited to share their perspectives on the impact of the pandemic on socially vulnerable adolescents. All interviews and focus groups were recorded, then fully transcribed and anonymised before being thematically analysed.

### Patient and public involvement

While patients and the public were not directly involved in the design, conduct, reporting or dissemination plans of the SEROCoV-KIDS study, we included open-ended questions at the end of each survey, asking participants to provide comments or suggest topics for future questionnaires. An interactive webinar was organised in February 2022, offering participants and the wider public the opportunity to ask questions to experts. The webinar is available for replay (https://www.youtube.com/watch?v=kP6PelXh1LM). Our group sends newsletters three to four times a year and maintains a website to keep participants and the general public informed about our studies. Our website features a news feed where study findings are presented in plain language (https://www.specchio-hub.ch/actualites). We also offer phone and email support for technical or study-related questions to encourage ongoing engagement.

## Findings to date

### Cohort characteristics

A total of 2199 children and adolescents from 1340 households were enrolled in the SEROCoV-KIDS cohort, 2048 in the population-based sample and 151 in the clinical sample (figure 1). The participation rate differed based on prior participation in seroprevalence studies (42.1% among previous participants and 9.1% among newly selected ones). Among new participants, older children and adolescents had higher participation rates (10.5% for ages 6–13 and 9.5% for ages 14–17) compared with children aged 1–5 years (7.1%). At enrolment, participants from the population-based sample were aged between 6 months and 17 years (mean: 9.8 years, SD: 4.3), with a balanced sex distribution (male: 49.8%; female: 50.1%; other: 0.1%; table 3). Most children were born in Switzerland (88.2%), had at least one parent with a tertiary education (83.8%) and lived in a household with a good or very good financial situation (81.5%). In the clinical sample, the mean age was 8.7 years (SD 4.8), and 46.4% were women. Children were mostly Swiss born (81.0%) and lived in households with highly educated parents (73.7%) and a good or very good financial situation (67.2%).

**Table 3:** Baseline characteristics of the 2199 children included in the SEROCoV-KIDS cohort (December 2021 to December 2022)

	General population sample		Clinical sample*	
	All participants (n=2048), n (%)	Adolescents only (n=525), n (%)	All participants (n=151), n (%)	Adolescents only (n=32), n (%)
Children characteristics				
Age at inclusion (years)				
0.5–5	389 (19.0)	0 (0.0)	48 (31.8)	0 (0.0)
6–13	1175 (57.4)	41 (7.8)†	71 (47.0)	0 (0.0)
14–17	484 (23.6)	484 (92.2)	32 (21.2)	32 (100.0)
Sex				
Female	1026 (50.1)	269 (51.2)	70 (46.4)	18 (56.3)
Male	1019 (49.8)	255 (48.6)	80 (53.0)	13 (40.6)
Other	3 (0.1)	1 (0.2)	1 (0.7)	1 (3.1)
Country of birth				
Switzerland	1685 (88.2)	405 (84.7)	94 (81.0)	15 (71.4)
Missing	137	47	35	11
Siblings				
Yes	1718 (89.9)	441 (92.3)	97 (83.6)	18 (85.7)
Missing	137	41	35	11
Parent-reported diagnosed chronic condition				
None	1350 (70.7)	285 (59.7)	24 (20.7)	4 (19.0)
Any physical condition	346 (18.1)	107 (22.4)	66 (56.9)	9 (42.9)
Any mental condition	146 (7.6)	54 (11.3)	7 (6.0)	2 (9.5)
Both	69 (3.6)	31 (6.5)	19 (16.4)	6 (28.6)
Missing	137	48	35	11
SARS-CoV-2 infection				
Positive serology (anti-SARS-CoV-2 N antibodies)	1264 (66.3)	293 (57.7)	105 (91.3)	15 (71.4)
Missing	142	17	36	11
Parents characteristics				
Referent parent				
Mother	1531 (80.1)	390 (81.6)	97 (83.6)	18 (85.7)
Missing	137	47	35	11
Single parenthood				
Yes	101 (5.6)	34 (7.5)	11 (10.3)	5 (29.4)
Missing	235	71	44	15
Highest educational level				
Tertiary	1602 (83.8)	393 (82.2)	84 (73.7)	14 (66.7)
Secondary	281 (14.7)	77 (16.1)	23 (20.2)	6 (28.6)
Primary	28 (1.5)	8 (1.7)	7 (6.1)	1 (4.8)
Missing	137	47	37	11
Household financial situation				
Very good	680 (37.8)	168 (37.4)	33 (30.8)	8 (42.1)
Good	785 (43.7)	203 (45.2)	39 (36.4)	2 (10.5)
Average to poor	333 (18.5)	78 (17.4)	35 (32.7)	9 (47.4)
Missing	250	76	44	13

Values are presented as numbers (%). Percentages calculated after excluding missing values.

* Clinical sample: children with chronic health conditions recruited by specialist physicians at the Children’s Hospital.

† Participants who turned 14 during the enrolment period and were invited to complete the baseline adolescent questionnaire.

SARS-CoV-2, Severe Acute Respiratory Syndrome Coronavirus 2.

### Attrition

Overall, questionnaire completion rates up to 2025 were 92.2% at baseline (2027/2199), followed by 76.9% (1692/2199), 66.8% (1464/2193), 60.8% (1302/2140), 55.6% (1190/2140) and 51.0% (1089/2134) at subsequent assessments, after excluding study withdrawals (table 4). For adolescent questionnaires, completion rates were 80.4% at baseline (448/557), and 61.6% (343/557), 63.7% (354/556), 52.4% (311/593), 49.9% (296/593) and 46.8% (276/590) at the following assessments (variations in the denominators are due to study withdrawals and the inclusion of adolescents who turned 14). Non-response was more frequent for older, non-Swiss children and those from single-parent families or households with average-to-poor financial situations (tables 4–6). As of fall 2025, a total of 70 (3.2%) parents and 31 adolescents (5.6%) had withdrawn from the study.

**Table 4:** Characteristics of overall cohort participants by response to follow-up questionnaires

	Baseline*(n=2199/2027)	Thematic questionnaire I (n=1692)			Annual questionnaire I (n=1464)			Thematic questionnaire II (n=1302)			Annual questionnaire II (n=1190)			Annual questionnaire III (n=1089)		
	Complete, n (%)	Complete, n (%)	Incomplete, n (%)	P value	Complete, n (%)	Incomplete, n (%)	P value	Complete, n (%)	Incomplete, n (%)	P value	Complete, n (%)	Incomplete, n (%)	P value	Complete, n (%)	Incomplete, n (%)	P value
Children characteristics																
Age at inclusion (years) (n=2199)																
0.5–5	437 (19.9)	345 (20.4)	92 (18.1)	<0.001	296 (20.2)	141 (19.2)	<0.001	267 (20.5)	170 (19.0)	<0.001	244 (20.5)	193 (19.1)	<0.001	239 (21.9)	198 (17.8)	<0.001
6–13	1246 (56.7)	1018 (60.2)	228 (45.0)		861 (58.8)	385 (52.4)		769 (59.1)	477 (53.2)		725 (60.9)	521 (51.6)		651 (59.8)	595 (53.6)	
14–17	516 (23.5)	329 (19.4)	187 (36.9)		307 (21.0)	209 (28.4)		266 (20.4)	250 (27.9)		221 (18.6)	295 (29.2)		199 (18.3)	317 (28.6)	
Sex (n=2199)																
Female	1096 (49.8)	833 (49.2)	263 (51.9)	0.575	733 (50.1)	363 (49.4)	0.206	653 (50.2)	443 (49.4)	0.053	601 (50.5)	495 (49.1)	0.415	554 (50.9)	542 (48.8)	0.405
Male	1099 (50.0)	856 (50.6)	243 (47.9)		730 (49.9)	369 (50.2)		649 (49.8)	450 (50.2)		588 (49.4)	511 (50.6)		534 (49.0)	565 (50.9)	
Other	4 (0.2)	3 (0.2)	1 (0.2)		1 (0.1)	3 (0.4)		0 (0.0)	4 (0.4)		1 (0.1)	3 (0.3)		1 (0.1)	3 (0.3)	
Country of birth (n=2027)																
Switzerland	1779 (87.8)	1488 (88.4)	291 (84.8)	0.085	1276 (89.4)	503 (84.0)	0.001	1138 (89.7)	641 (84.6)	0.001	1047 (90.2)	732 (84.5)	<0.001	975 (91.1)	804 (84.0)	<0.001
Siblings (n=2027)																
Yes	1815 (89.5)	1490 (88.5)	325 (94.8)	0.001	1267 (88.7)	548 (91.5)	0.076	1120 (88.3)	695 (91.7)	0.018	1026 (88.4)	789 (91.1)	0.055	940 (87.9)	875 (91.4)	0.011
Diagnosed chronic condition (n=2027)																
None	1374 (67.8)	1143 (67.9)	231 (67.5)	0.809	951 (66.6)	423 (70.6)	0.218	855 (67.4)	519 (68.5)	0.461	808 (69.7)	566 (65.4)	0.113	736 (68.8)	638 (66.7)	0.196
Any physical condition	412 (20.3)	345 (20.5)	67 (19.6)		302 (21.2)	110 (18.4)		270 (21.3)	142 (18.7)		230 (19.8)	182 (21.0)		222 (20.8)	190 (19.9)	
Any mental condition	153 (7.6)	123 (7.3)	30 (8.8)		107 (7.5)	46 (7.7)		92 (7.3)	61 (8.0)		76 (6.6)	77 (8.9)		70 (6.5)	83 (8.7)	
Both	88 (4.3)	73 (4.3)	14 (4.1)		67 (4.7)	20 (3.3)		51 (4.0)	36 (4.7)		46 (4.0)	41 (4.7)		41 (3.8)	46 (4.8)	
SARS-CoV-2 infection (n=2021)																
Positive serology	1369 (67.7)	1052 (67.3)	317 (69.2)	0.610	894 (65.9)	475 (71.5)	0.035	809 (66.9)	560 (69.1)	0.529	741 (67.2)	628 (68.3)	0.316	669 (66.6)	700 (68.9)	0.295
Parents characteristics																
Mother referent parent (n=2027)	1628 (80.3)	1344 (79.8)	284 (82.8)	0.419	1156 (81.0)	472 (78.8)	0.100	1011 (79.7)	617 (81.4)	0.266	926 (79.8)	702 (81.1)	0.872	863 (80.7)	765 (79.9)	0.640
Single parenthood (n=1920)	112 (5.8)	89 (5.5)	23 (7.3)	0.278	73 (5.3)	39 (7.1)	0.171	55 (4.5)	57 (8.2)	0.001	58 (5.2)	54 (6.8)	0.156	52 (5.0)	60 (6.8)	0.124
Highest educational level (n=2025)																
Tertiary	1686 (83.3)	1399 (83.2)	287 (83.7)	0.824	1203 (84.2)	483 (80.9)	0.177	1078 (84.9)	608 (80.4)	0.025	991 (85.4)	695 (80.4)	0.013	929 (86.8)	757 (79.3)	<0.001
Secondary	304 (15.0)	255 (15.2)	49 (14.3)		201 (14.1)	103 (17.3)		173 (13.6)	131 (17.3)		152 (13.1)	152 (17.6)		131 (12.2)	173 (18.1)	
Primary	35 (1.7)	28 (1.7)	7 (2.0)		24 (1.7)	11 (1.8)		18 (1.4)	17 (2.2)		18 (1.6)	17 (2.0)		10 (0.9)	25 (2.6)	
Household financial situation (n=1905)																
Very good	713 (37.4)	602 (37.8)	111 (35.7)	0.063	513 (37.7)	200 (36.8)	0.149	489 (40.3)	224 (32.3)	0.001	429 (38.6)	284 (35.7)	0.313	419 (41.0)	294 (33.3)	0.001
Good	824 (43.3)	699 (43.9)	125 (40.2)		600 (44.1)	224 (41.2)		510 (42.1)	314 (45.3)		477 (43.0)	347 (43.6)		428 (41.9)	396 (44.8)	
Average to poor	368 (19.3)	293 (18.4)	75 (24.1)		248 (18.2)	120 (22.1)		213 (17.6)	155 (22.4)		204 (18.4)	164 (20.6)		174 (17.0)	194 (21.9)	
SEROCoV-KIDS sample (n=2199)																
General population	2048 (93.1)	1578 (93.3)	470 (92.7)	0.736	1368 (93.4)	680 (92.5)	0.471	1213 (93.2)	835 (93.1)	1.000	1123 (94.4)	925 (91.7)	0.016	1028 (94.4)	1020 (91.9)	0.025
Clinical	151 (6.9)	114 (6.7)	37 (7.3)		96 (6.6)	55 (7.5)		89 (6.8)	62 (6.9)		67 (5.6)	84 (8.3)		61 (5.6)	90 (8.1)	

* At baseline, age and sex data were available for all 2199 participants and 2027 questionnaires were fully completed.

**Table 5:** Characteristics of participants from the general population sample by response to follow-up questionnaires

	Baseline* (n=2048/1911)	Thematic questionnaire I (n=1578)			Annual questionnaire I (n=1368)			Thematic questionnaire II (n=1213)			Annual questionnaire II (n=1123)			Annual questionnaire III (n=1028)		
	Complete, n (%)	Complete, n (%)	Incomplete, n (%)	P value	Complete, n (%)	Incomplete, n (%)	P value	Complete, n (%)	Incomplete, n (%)	P value	Complete, n (%)	Incomplete, n (%)	P value	Complete, n (%)	Incomplete, n (%)	P value
Children characteristics																
Age at inclusion (years) (n=2048)																
0.5–5	389 (19.0)	311 (19.7)	78 (16.6)	<0.001	261 (19.1)	128 (18.8)	0.004	239 (19.7)	150 (18.0)	0.001	225 (20.0)	164 (17.7)	<0.001	216 (21.0)	173 (17.0)	<0.001
6–13	1175 (57.4)	961 (60.9)	214 (45.5)		813 (59.4)	362 (53.2)		724 (59.7)	451 (54.0)		687 (61.2)	488 (52.8)		621 (60.4)	554 (54.3)	
14–17	484 (23.6)	306 (19.4)	178 (37.9)		294 (21.5)	190 (27.9)		250 (20.6)	234 (28.0)		211 (18.8)	273 (29.5)		191 (18.6)	293 (28.7)	
Sex (n=2048)																
Female	1026 (50.1)	781 (49.5)	245 (52.1)	0.400	687 (50.2)	339 (49.9)	0.466	614 (50.6)	412 (49.3)	0.100	569 (50.7)	457 (49.4)	0.653	527 (51.3)	499 (48.9)	0.492
Male	1019 (49.8)	794 (50.3)	225 (47.9)		680 (49.7)	339 (49.9)		599 (49.4)	420 (50.3)		553 (49.2)	466 (50.4)		500 (48.6)	519 (50.9)	
Other	3 (0.1)	3 (0.2)	0 (0.0)		1 (0.1)	2 (0.3)		0 (0.0)	3 (0.4)		1 (0.1)	2 (0.2)		1 (0.1)	2 (0.2)	
Country of birth (n=1911)																
Switzerland	1685 (88.2)	1402 (88.9)	283 (84.7)	0.040	1200 (89.8)	485 (84.3)	0.001	1070 (90.1)	615 (84.9)	0.001	994 (90.4)	691 (85.1)	<0.001	924 (91.4)	761 (84.6)	<0.001
Siblings (n=1911)																
Yes	1718 (89.9)	1401 (88.8)	317 (94.9)	0.001	1192 (89.2)	526 (91.5)	0.156	1052 (88.6)	666 (92.0)	0.022	973 (88.5)	745 (91.7)	0.026	891 (88.1)	827 (91.9)	0.008
Diagnosed chronic condition (n=1911)																
None	1350 (70.7)	1121 (71.1)	229 (68.8)	0.645	934 (70.0)	416 (72.3)	0.592	838 (70.7)	512 (70.7)	0.762	796 (72.5)	554 (68.2)	0.077	725 (71.8)	625 (69.4)	0.235
Any physical condition	346 (18.1)	286 (18.1)	60 (18.0)		249 (18.7)	97 (16.9)		221 (18.6)	125 (17.3)		196 (17.9)	150 (18.5)		186 (18.4)	160 (17.8)	
Any mental condition	146 (7.6)	116 (7.4)	30 (9.0)		101 (7.6)	45 (7.8)		87 (7.3)	59 (8.1)		72 (6.6)	74 (9.1)		67 (6.6)	79 (8.8)	
Both	69 (3.6)	54 (3.4)	14 (4.2)		51 (3.8)	17 (3.0)		40 (3.4)	28 (3.9)		34 (3.1)	34 (4.2)		32 (3.2)	36 (4.0)	
SARS-CoV-2 infection (n=1906)																
Positive serology	1264 (66.3)	972 (65.9)	292 (67.9)	0.592	827 (64.3)	437 (70.5)	0.025	743 (65.2)	521 (67.9)	0.432	692 (65.9)	572 (66.8)	0.315	626 (65.3)	638 (67.4)	0.336
Parents characteristics																
Mother referent parent (n=1911)	1531 (80.1)	1256 (79.6)	275 (82.3)	0.460	1077 (80.6)	454 (79.0)	0.327	940 (79.2)	591 (81.6)	0.379	874 (79.5)	657 (80.9)	0.887	812 (80.3)	719 (79.9)	0.907
Single parenthood (n=1813)	101 (5.6)	78 (5.2)	23 (7.5)	0.141	66 (5.1)	35 (6.6)	0.268	48 (4.2)	53 (8.0)	0.001	52 (4.9)	49 (6.6)	0.145	48 (4.9)	53 (6.4)	0.215
Highest educational level (n=1911)																
Tertiary	1602 (83.8)	1320 (83.7)	282 (84.4)	0.756	1130 (84.6)	472 (82.1)	0.381	1015 (85.5)	587 (81.1)	0.013	942 (85.7)	660 (81.3)	0.034	877 (86.7)	725 (80.6)	<0.001
Secondary	281 (14.7)	235 (14.9)	46 (13.8)		188 (14.1)	93 (16.2)		160 (13.5)	121 (16.7)		143 (13.0)	138 (17.0)		126 (12.5)	155 (17.2)	
Primary	28 (1.5)	22 (1.4)	6 (1.8)		18 (1.3)	10 (1.7)		12 (1.0)	16 (2.2)		14 (1.3)	14 (1.7)		8 (0.8)	20 (2.2)	
Household financial situation (n=1798)																
Very good	680 (37.8)	571 (38.2)	109 (35.9)	0.016	483 (38.0)	197 (37.5)	0.221	462 (40.7)	218 (32.8)	0.002	406 (38.7)	274 (36.6)	0.599	398 (41.3)	282 (33.8)	0.002
Good	785 (43.7)	664 (44.4)	121 (39.8)		566 (44.5)	219 (41.6)		481 (42.4)	304 (45.8)		456 (43.4)	329 (44.0)		407 (42.2)	378 (45.3)	
Average to poor	333 (18.5)	259 (17.3)	74 (24.3)		223 (17.5)	110 (20.9)		191 (16.8)	142 (21.4)		188 (17.9)	145 (19.4)		159 (16.5)	174 (20.9)	

* At baseline, age and sex data were available for all 2048 participants and 1911 questionnaires were fully completed.

**Table 6:** Characteristics of participants from the clinical sample (children with chronic health conditions) by response to follow-up questionnaires

	Baseline* (n=151/116)	Thematic questionnaire I (n=114)			Annual questionnaire I (n=96)			Thematic questionnaire II (n=89)			Annual questionnaire II (n=67)			Annual questionnaire III (n=61)		
	Complete, n (%)	Complete, n (%)	Incomplete, n (%)	P value	Complete, n (%)	Incomplete, n (%)	P value	Complete, n (%)	Incomplete, n (%)	P value	Complete, n (%)	Incomplete, n (%)	P value	Complete, n (%)	Incomplete, n (%)	P value
Children characteristics																
Age at inclusion (years) (n=151)																
0.5–5	48 (31.8)	34 (29.8)	14 (37.8)	0.434	35 (36.5)	13 (23.6)	0.008	28 (31.5)	20 (32.3)	0.440	19 (28.4)	29 (34.5)	0.079	23 (37.7)	25 (27.8)	0.112
6–13	71 (47.0)	57 (50.0)	14 (37.8)		48 (50.0)	23 (41.8)		45 (50.6)	26 (41.9)		38 (56.7)	33 (39.3)		30 (49.2)	41 (45.6)	
14–17	32 (21.2)	23 (20.2)	9 (24.3)		13 (13.5)	19 (34.5)		16 (18.0)	16 (25.8)		10 (14.9)	22 (26.2)		8 (13.1)	24 (26.7)	
Sex (n=151)																
Female	70 (46.4)	52 (45.6)	18 (48.6)	0.191	46 (47.9)	24 (43.6)	0.382	39 (43.8)	31 (50.0)	0.340	32 (47.8)	38 (45.2)	0.650	27 (44.3)	43 (47.8)	0.631
Male	80 (53.0)	62 (54.4)	18 (48.6)		50 (52.1)	30 (54.5)		50 (56.2)	30 (48.4)		35 (52.2)	45 (53.6)		34 (55.7)	46 (51.1)	
Other	1 (0.7)	0 (0.0)	1 (2.7)		0 (0.0)	1 (1.8)		0 (0.0)	1 (1.6)		0 (0.0)	1 (1.2)		0 (0.0)	1 (1.1)	
Country of birth (n=116)																
Switzerland	94 (81.0)	86 (80.4)	8 (88.9)	0.855	76 (82.6)	18 (75.0)	0.579	68 (82.9)	26 (76.5)	0.584	53 (85.5)	41 (75.9)	0.284	51 (86.4)	43 (75.4)	0.203
Siblings (n=116)																
Yes	97 (83.6)	89 (83.2)	8 (88.9)	1.000	75 (81.5)	22 (91.7)	0.375	68 (82.9)	29 (85.3)	0.970	53 (85.5)	44 (81.5)	0.742	49 (83.1)	48 (84.2)	1.000
Diagnosed chronic condition (n=116)																
None	24 (20.7)	22 (20.6)	2 (22.2)	0.400	17 (18.5)	7 (29.2)	0.672	17 (20.7)	7 (20.6)	0.592	12 (19.4)	12 (22.2)	0.808	11 (18.6)	13 (22.8)	0.832
Any physical condition	66 (56.9)	59 (55.1)	7 (77.8)		53 (57.6)	13 (54.2)		49 (59.8)	17 (50.0)		34 (54.8)	32 (59.3)		36 (61.0)	30 (52.6)	
Any mental condition	7 (6.0)	7 (6.5)	0 (0.0)		6 (6.5)	1 (4.2)		5 (6.1)	2 (5.9)		4 (6.5)	3 (5.6)		3 (5.1)	4 (7.0)	
Both	19 (16.4)	19 (17.8)	0 (0.0)		16 (17.4)	3 (12.5)		11 (13.4)	8 (23.5)		12 (19.4)	7 (13.0)		9 (15.3)	10 (17.5)	
SARS-CoV-2 infection (n=115)																
Positive serology	105 (91.3)	80 (92.0)	25 (89.3)	0.960	67 (94.4)	38 (86.4)	0.254	66 (93.0)	39 (88.6)	0.646	49 (94.2)	56 (88.9)	0.497	43 (93.5)	62 (89.9)	0.736
Parents characteristics																
Mother referent parent (n=116)	97 (83.6)	88 (82.2)	9 (100.0)	0.591	79 (85.9)	18 (75.0)	0.161	71 (86.6)	26 (76.5)	0.233	52 (83.9)	45 (83.3)	0.568	51 (86.4)	46 (80.7)	0.431
Single parenthood (n=107)	11 (10.3)	11 (11.1)	0 (0.0)	0.696	7 (8.0)	4 (20.0)	0.238	7 (9.2)	4 (12.9)	0.826	6 (10.3)	5 (10.2)	1.000	4 (7.1)	7 (13.7)	0.423
Highest educational level (n=114)																
Tertiary	84 (73.7)	79 (75.2)	5 (55.6)	0.434	73 (79.3)	11 (50.0)	0.004	63 (76.8)	21 (65.6)	0.153	49 (79.0)	35 (67.3)	0.258	52 (88.1)	32 (58.2)	0.001
Secondary	23 (20.2)	20 (19.0)	3 (33.3)		13 (14.1)	10 (45.5)		13 (15.9)	10 (31.2)		9 (14.5)	14 (26.9)		5 (8.5)	18 (32.7)	
Primary	7 (6.1)	6 (5.7)	1 (11.1)		6 (6.5)	1 (4.5)		6 (7.3)	1 (3.1)		4 (6.5)	3 (5.8)		2 (3.4)	5 (9.1)	
Household financial situation (n=107)																
Very good	33 (30.8)	31 (31.0)	2 (28.6)	0.434	30 (33.7)	3 (16.7)	0.070	27 (34.6)	6 (20.7)	0.206	23 (38.3)	10 (21.3)	0.129	21 (36.8)	12 (24.0)	0.228
Good	39 (36.4)	35 (35.0)	4 (57.1)		34 (38.2)	5 (27.8)		29 (37.2)	10 (34.5)		21 (35.0)	18 (38.3)		21 (36.8)	18 (36.0)	
Average to poor	35 (32.7)	34 (34.0)	1 (14.3)		25 (28.1)	10 (55.6)		22 (28.2)	13 (44.8)		16 (26.7)	19 (40.4)		15 (26.3)	20 (40.0)	

* At baseline, age and sex data were available for all 151 participants, and 116 questionnaires were fully completed.

### Prevalence of anti-SARS-CoV-2 antibodies and the health of children and adolescents with prior SARS-CoV-2 infection

The baseline visit coincided with the widespread circulation of the Omicron variant, which led to a high infection rate among the paediatric population. As a result, the majority of children and adolescents tested seropositive for anti-SARS-CoV-2 N antibodies, reaching 66.3% in the population-based sample recruited between December 2021 and June 2022 and 91.3% in the clinical sample recruited between June and December 2022 (tables 5 and 6). Among them, 31.1% and 58.6% reported having had a positive PCR or rapid antigenic test. Two years into the pandemic, a substantial number of children and adolescents experienced symptoms consistent with post-COVID-19 condition as defined by the WHO in March 2022, that is, persistent symptoms lasting more than 12 weeks after a confirmed SARS-CoV-2 infection and without an alternative diagnosis.^54^ In the population-based sample, the overall prevalence of paediatric post-COVID-19 condition was estimated to be 4.1% (95% CI 1.1 to 7.3), with adolescents showing a significantly higher prevalence (8.3%, 95% CI 3.5 to 13.5).^55^ The main risk factors identified for post-COVID-19 condition included older age, disadvantaged socioeconomic conditions and the presence of a chronic health condition, particularly asthma. We further explored a potential pathophysiological pathway linking acute SARS-CoV-2 infection to persistent symptoms. Our findings suggest that SARS-CoV-2 infection induces the production of autoantibodies against apolipoprotein A-1 (AAA1), which are associated with short-term symptom persistence in children, corroborating previous observations in adults.^56^

### Health and psychosocial consequences of the pandemic in the general paediatric population

In 2021–2022, at baseline, four out of five children had not been impacted by the pandemic or only minimally, and displayed low levels of mental health difficulties throughout the 2022–2025 period.^57 58^ This overall reassuring finding suggests that Switzerland’s relatively light restrictions and broad social benefits helped mitigate the pandemic’s health impact on children. Approximately 8% of participants reported a positive pandemic impact. Their baseline mental health and evolution over time were comparable to those of participants who experienced no impact. Conversely, depending on the measurement tool, 8%–13% of children and their households experienced a pronounced negative impact, mainly due to disruptions in daily routines and reduced access to social and family support.^57 58^ They exhibited poorer health-related quality of life (HRQoL) and mental health in 2022.^57 58^ Although mental health improved in this group between 2022 and 2025, it remained poorer than among children who were not impacted.^58^ While these findings are reassuring overall, they underscore that the pandemic’s effects on youth well-being persisted for years, even after most restrictions had been lifted.

Since many families reported challenges in balancing work and childcare early in the pandemic, we investigated whether parents’ work-to-family conflict was associated with trajectories of mental health for both parents and children between 2022 and 2024.^59^ Higher levels of conflict were consistently associated with worse anxiety and depressive symptoms in parents, as well as with internalising and externalising difficulties in children.

We further explored specific aspects of the mental health of adolescents. This developmental stage is characterised by profound physical, psychological and social changes that significantly influence the transition into adulthood.^60^ Each generation of adolescents faces unique contextual challenges, such as the pandemic and related restrictions, adding complexity to their developmental trajectory. In 2022, we observed that 14% of adolescents in the population-based sample reported suicidal ideation in the previous year, a rate similar to global pre-pandemic levels.^61^ Key risk factors included psychological distress, low self-esteem, identification with a sexual minority, bullying, excessive screen time and a severe pandemic impact. We also examined the psychological burden of acne, finding that adolescents with lower acne-specific quality of life experienced greater distress, lower self-esteem and reduced resilience.^62^ These effects were mitigated by physical activity and social support, but worsened by screen time and social media addiction. Lastly, in another study we reported that one in six adolescents felt limited in their future perspectives, and this was particularly true for those with mental health difficulties, limited social support, academic struggles, sexual minority identity and excessive screen time. Major concerns were centred on failure, education and climate change.^63^ In adolescents, many risk factors identified are interrelated and contribute to multiple mental health challenges, underscoring the need for integrated, multifaceted approaches to prevention and intervention. Conversely, strong parent-child relationships, social support, physical activity and high self-esteem emerged as key protective factors across various domains of mental health.^61–63^

Health behaviours are essential to the physical and mental health of children and adolescents.^64^ Prolonged pandemic control measures, such as remote schooling, limitation of in-person interactions and restricted access to recreational facilities, disrupted the health behaviours of young people. In Switzerland, however, these restrictions were comparatively milder than in many neighbouring countries.^65^ Our findings show that, 2 years into the pandemic, 20%–30% of children failed to meet health behaviour recommendations for physical activity, screen time and sleep duration.^66^ For most children, physical activity (72.9%), time spent in green spaces (72.2%) and sleep quality (93.1%) remained unchanged throughout the pandemic, while over half (51.9%) reported increased screen time. This increase in screen time is concerning given the observed association between screen time and lower quality of life,^62 67^ psychological distress^61^ or future perspectives^63^ among preadolescents and adolescents.

### Health and psychosocial consequences of the pandemic in children with chronic health conditions

Children living with chronic health conditions or having special healthcare needs face greater challenges to mental health and quality of life compared with the general paediatric population.^68–71^ Our findings showed that, although these children were not at higher risk of SARS-CoV-2 infection compared with their healthy peers, they were more likely to suffer from persistent symptoms^55^ and to report a severe pandemic impact.^57 72^ After pandemic-related restrictions were lifted, children with special healthcare needs had poorer well-being than healthy children, with a gradient according to the complexity of their condition.^72^ Children with chronic conditions also experienced greater difficulties in physical and social functioning and externalising behaviours, but reported similar levels of prosocial behaviour, social support and extracurricular participation as their healthy peers. Those with complex chronic conditions were at particularly high risk for poor well-being across physical, psychological and social domains. Even after restrictions ended, only a few well-being aspects improved, while many others worsened.^72^ These results highlight the need for long-term follow-up of post-pandemic well-being and targeted interventions to support these children.

### Health and psychosocial consequences of the pandemic in socioeconomically disadvantaged populations

Several of our studies have highlighted how the pandemic exacerbated existing social inequalities in health. Socioeconomic and family disadvantage was consistently associated with a more severe pandemic impact^57 73^ and greater psychosocial difficulties (having few close friends, school difficulties, no engagement in extracurricular activities).^74 75^ Disadvantaged children were also more likely to engage in unhealthy behaviours (not meeting sleep duration guidelines, limited physical activity, smoking, soda consumption, low adherence to COVID-19 vaccination guidelines)^66 74 76^ and were more frequently affected by a range of unfavourable health outcomes, including persistent symptoms compatible with post-COVID-19 condition,^55^ poor HRQoL,^74 77^ internalising and externalising difficulties,^77^ poorer well-being and higher anxiety at school.^75^ Our results also suggest that certain psychosocial factors, such as a good parent-child relationship or social support, can act as protective factors for poor outcomes.^61 62 74^

Disadvantaged children showed a milder mental health response to the negative impact of the pandemic than advantaged children, and mental health difficulties decreased over time in both groups, regardless of the financial situation of their households.^73^ This illustrates the complexity of the pandemic’s social impact and suggests that, while disadvantaged children were more vulnerable to pandemic-related burdens, many demonstrated coping resources and notable resilience.

The qualitative PIRA study further illuminated this complexity by capturing the experiences of 17 adolescents and 20 professionals working with adolescents.^53^ It highlighted the differentiated effects of the health crisis on young people, especially the conflicting expectations they faced across their school, family and social roles, which they had to adopt, abandon and relearn. The shift to remote learning exacerbated educational inequalities, making reintegration more challenging for vulnerable youth, sometimes leading to disengagement. Family life had contrasting effects: while some households experienced strengthening bonds, others, particularly those in precarious conditions, faced increased tensions. Social isolation, especially among certain groups such as girls, was partly offset by intensified online interactions, with mixed consequences. The return to normalcy revealed and exacerbated social and psychological stress. This study component showed that the most vulnerable youth accumulated difficulties during both the acute phase of the pandemic and the return to normalcy, widening social disparities with potentially lasting effects on their trajectories. It emphasised the need to analyse these experiences within relational dynamics between peers and adults, reflecting the quantitative findings of the cohort.

## Strengths and limitations

The SEROCoV-KIDS study is one of the few paediatric cohorts established during the pandemic. Its main strengths include its population-based design with random sampling, its large sample size, the broad age range covered, its longitudinal follow-up and the use of a combination of quantitative and qualitative approaches, all of which enhance the external validity and relevance of the findings. However, several limitations should be considered when interpreting our findings. First, the study is susceptible to information bias because the lack of individual-level pre-pandemic and early-pandemic data limits direct comparisons of changes in children’s health, well-being and behaviours over time. Instead, the study relies on parent-reported assessments of the pandemic’s impact which may be affected by recall bias. Consequently, robust conclusions regarding temporal trends can only be drawn from repeated measures collected between 2021 and 2025. Nevertheless, these findings still provide valuable insights given the lack of a national cohort of children in Switzerland. Second, despite random sampling, selection bias led to the overrepresentation of children aged 8–14, because parents were often reluctant to have younger children undergo a blood draw solely for research purposes and because adolescents had to complete their own questionnaires, which might have reduced their willingness to participate. As commonly observed in epidemiological studies, highly educated families were also overrepresented in the cohort, likely reflecting greater social engagement and trust in science among more privileged groups.^78^ This may limit the generalisability of our findings and could lead to an overestimation of population-level well-being and health behaviours. Additionally, even though one-fifth of the cohort reported financial difficulties, the most disadvantaged children were likely underrepresented, potentially leading to an underestimation of adverse outcomes in populations facing the greatest pandemic-related challenges. As with all observational studies, residual confounding cannot be excluded. Although the rich longitudinal data collected in the cohort allow adjustment for a wide range of sociodemographic, health and family characteristics, unmeasured or imperfectly measured factors may still influence observed associations. Finally, despite various incentives to maintain participation, the study experienced ‘COVID fatigue’, resulting in substantial attrition over time. Because loss to follow-up was more frequent among older, non-Swiss children and those from socioeconomically disadvantaged backgrounds, these groups may be underrepresented in later follow-up waves, potentially leading to an underestimation of social inequalities and of the long-term impact of the pandemic on vulnerable populations.

## Collaboration

We welcome collaborative projects, and researchers may apply for access to the SEROCoV-KIDS biobank and cohort data, subject to authorisation from our scientific board and ethics approval for their study. Requests for study questionnaires and data access can be submitted to uep@hug.ch.

## Conclusions and future plans

The SEROCoV-KIDS cohort demonstrates that sustained, population-based paediatric research is essential for monitoring and understanding how public policies and major societal events shape child and adolescent health, and for informing evidence-based decision-making. As a uniquely large paediatric cohort study in Switzerland, SEROCoV-KIDS strengthens the evidence base on how the pandemic and its aftermath have affected children and adolescents, revealing both resilience and persistent inequalities.

Its findings underscore the need to invest in long-term, population-based cohort infrastructures capable of generating timely, multidimensional data that integrate biological, psychological and social dimensions. Such sustained monitoring systems are essential for detecting early warning signals, evaluating interventions and designing preventive strategies that promote health equity and well-being. More than a scientific imperative, this investment is a public responsibility.

Future work will focus on further exploiting the rich longitudinal data and biobank resources collected through the cohort. Planned analyses include investigations of environmental exposures and their associations with child and adolescent health outcomes, as well as studies examining the long-term consequences of the COVID-19 pandemic on health, well-being and social inequalities. The biobank provides unique opportunities to explore biological and environmental determinants of health and to address emerging research questions that were not anticipated at the time of cohort inception.

Subject to future funding, continued follow-up of participants would provide valuable insights into the long-term trajectories of health and well-being across childhood, adolescence and young adulthood. Findings from SEROCoV-KIDS will continue to be shared with researchers, healthcare professionals and public health authorities to support evidence-informed policies and interventions aimed at improving child and adolescent health.

More broadly, SEROCoV-KIDS provides a model for anticipatory, evidence-informed governance that shifts societies from crisis response towards building environments that protect, empower and support future generations, while also offering, subject to future funding, a valuable platform for rapidly assessing the impact of future pandemics, public health emergencies and other major societal disruptions on child and adolescent health, well-being and inequalities.

## Data Availability

Data are available upon reasonable request.
