# Supplementary material for: Cohort profile: the SEROCoV-KIDS population-based cohort study and biobank on the impact of the COVID-19 pandemic in children and adolescents in Geneva, Switzerland
Source: BMJ Open. 2026 Sep 21;16(9):e116875. doi: 10.1136/bmjopen-2026-116875 (PMC13599830; doi:10.1136/bmjopen-2026-116875)
Supplement: Supplementary file 1 [file bmjopen-2026-116875.reviewer_comments.pdf]

PEER REVIEW HISTORY

BMJ Open publishes all reviews undertaken for accepted manuscripts. Reviewers are asked to complete a checklist review form and are provided with free text boxes to elaborate on their assessment. These free text comments are reproduced below.

ARTICLE DETAILS

Title (Provisional)

Cohort profile: the SEROCoV-KIDS population-based cohort study and biobank on the impact of the COVID-19 pandemic in children and adolescents in Geneva, Switzerland

Authors

Lorthe, Elsa; Loizeau, Andrea; Richard, Viviane; Dumont, Roxane; Zaballa, María-Eugenia; Pennacchio, Francesco; Lamour, Julien; L'huillier, Arnaud G; Baysson, Hélène; Fernandez Clares, Natalia; Bovio, Nicolas; Nehme, Mayssam; Lescuyer, Pierre; Vuilleumier , Nicolas; Posfay-Barbe, Klara M; Barbe, Rémy P; study group, SEROCoV-KIDS study group; Guessous, Idris; Stringhini, Silvia

---

VERSION 1 - REVIEW

**Reviewer**	**1**
**Name**	**Tung, Tao-Hsin**
**Affiliation**	**Taizhou Hospital of Zhejiang Province, Evidence-based Medicine Center**
**Date**	**20-May-2026**
**COI**	**None**

This SEROCoV-KIDS cohort profile is well-structured, methodologically rigorous, and data-rich, providing high-quality evidence on the short- and long-term impacts of the COVID-19 pandemic on children and adolescents in Geneva. With a large population-based sample, long-term longitudinal follow-up, integrated quantitative and qualitative data, and a dedicated biobank, the study makes a substantial contribution to pediatric public health research and policy. The ethical design, data management, and collaborative framework are strong and meet high academic standards. Several suggestions are as follows:

1. Pages 6-8. The introduction lacks logical flow between pandemic impacts, child vulnerability, and social inequalities. Add transitional sentences to strengthen coherence.

2. Pages 9-11. The description of the Geneva pandemic context is overly detailed. Simplify to 2-3 key policy milestones relevant to children.

3. Pages 11-12. Recruitment procedures for population and clinical samples are interleaved and unclear. Use separate subsections to improve readability. In addition, the PIRA qualitative study is poorly integrated. Use a distinct subheading to define its design, population, and methods clearly.

4. Pages 13-14. Attrition rates are reported but not interpreted. Add a brief analysis of reasons for dropout and potential bias.

5. Pages 18-19. The limitations section is underdeveloped. Systematically address selection bias, information bias, and confounding for transparency.

6. Pages 19-20. Future plans are vague. Specify planned research questions, indicators, surveillance strategies, and policy translation pathways.

**Reviewer**	**2**
**Name**	**Chaw, Liling**
**Affiliation**	**University of Brunei, PAPRSB Institute of Health Sciences**
**Date**	**20-May-2026**
**COI**	**None**

Thank you for the opportunity to review this cohort profile. Overall, this is a very well-written manuscript with clear rationale and aims. I congratulate all the co-authors for all the published work conducted from this cohort data, and also appreciate the willingness to collaborate with other interested researchers. I have a few comments that may further improve the manuscript.

(In pg.9, under Cohort study design, population and recruitment procedures)

1. Was sample size calculated to ensure representativeness of the results to the population?

2. Could you explain more on how random sampling was done from state registries?

3. Please provide the rationale for recruiting Children with chronic health conditions who were referred by specialist physicians working at the Children's Hospital (Geneva University Hospitals): Were these the only source of children with chronic health conditions in Geneva?

4. Suggest clarifying if the participants recruited in the pilot phase were also included in the main study.

5. On the future plans for this cohort, suggest to consider how the cohort can be continued as in the context of future COVID-like pandemics (if possible & if funding can be secured).

VERSION 1 - AUTHOR RESPONSE

Reviewer 1

This SEROCoV-KIDS cohort profile is well-structured, methodologically rigorous, and data-rich, providing high-quality evidence on the short- and long-term impacts of the COVID-19 pandemic on children and adolescents in Geneva. With a large population-based sample, long-term longitudinal follow-up, integrated quantitative and qualitative data, and a dedicated biobank, the study makes a substantial contribution to pediatric public health research and policy. The ethical design, data management, and collaborative framework are strong and meet high academic standards.
Thank you for the positive feedback, and for your comments which helped us improve the article.

Several suggestions are as follows:
1. Pages 6-8. The introduction lacks logical flow between pandemic impacts, child vulnerability, and social inequalities. Add transitional sentences to strengthen coherence.
In line with this judicious suggestion, we added transitional sentences at the beginning of the second and third paragraphs of the introduction to improve coherence and better articulate the progression from pandemic impacts to child vulnerability and social inequalities.

2. Pages 9-11. The description of the Geneva pandemic context is overly detailed. Simplify to 2-3 key policy milestones relevant to children.
We thank the reviewer for this suggestion. We shortened the description of the Geneva COVID-19 context and focused on the key policy milestones most relevant to children and adolescents, namely the initial school closures during the first wave, the subsequent prioritization of keeping schools open, the progressive lifting of restrictions in 2022, and the rollout of COVID-19 vaccination to children.

3. Pages 11-12. Recruitment procedures for population and clinical samples are interleaved and unclear. Use separate subsections to improve readability. In addition, the PIRA qualitative study is poorly integrated. Use a distinct subheading to define its design, population, and methods clearly.
We thank the reviewer for highlighting this. To improve readability, we reorganized the description of the cohort study recruitment procedures by creating separate paragraphs for the population-based sample and the clinical sample. We also revised the section heading related to the qualitative component to more clearly reflect its design, population, and methods. In addition, we expanded the description of the PIRA study by providing further details on the recruitment of frontline professionals who participated in the focus groups.

4. Pages 13-14. Attrition rates are reported but not interpreted. Add a brief analysis of reasons for dropout and potential bias.
We believe that the Findings section should focus on describing participant retention and attrition patterns rather than interpreting their implications. We already provide detailed information on questionnaire completion rates over time, study withdrawals, and participant characteristics associated with non-response. To address the reviewer's concern regarding the potential impact of attrition on study findings, we have added a statement in the Strengths and limitations section discussing the bias resulting from the differential loss to follow-up observed among socially disadvantaged participants.

5. Pages 18-19. The limitations section is underdeveloped. Systematically address selection bias, information bias, and confounding for transparency.

We thank the reviewer for this suggestion. The limitations section already discussed several sources of selection bias, including participation bias at recruitment, the underrepresentation of socioeconomically disadvantaged families, and differential loss to follow-up over time. It also addressed potential information bias arising from the use of parent-reported measures and retrospective assessments of the pandemic's impact. To improve transparency, we revised the section to explicitly frame these issues in terms of selection and information bias and added a statement acknowledging the possibility of residual confounding in future analyses of cohort data.

6. Pages 19-20. Future plans are vague. Specify planned research questions, indicators, surveillance strategies, and policy translation pathways.
Thank you for pointing out the need for clarification. We revised the "Conclusions and future plans" section to provide a more concrete description of the scientific opportunities offered by the cohort. In particular, we now highlight the planned exploitation of the longitudinal data and biobank resources, including studies on environmental exposures and child health outcomes, and clarify that continued participant follow-up and surveillance activities will depend on the availability of future funding. We also expanded the description of how findings may inform public health decision-making and future interventions.

Reviewer 2

Thank you for the opportunity to review this cohort profile. Overall, this is a very well-written manuscript with clear rationale and aims. I congratulate all the co-authors for all the published work conducted from this cohort data, and also appreciate the willingness to collaborate with other interested researchers. I have a few comments that may further improve the manuscript.
We would like to thank the reviewer for this positive evaluation of our work. These questions are very relevant, and we believe that clarifying these points will be very beneficial for the readers and for future collaborations.

(In pg.9, under Cohort study design, population and recruitment procedures)
1. Was sample size calculated to ensure representativeness of the results to the population?
We thank the reviewer for this question. The primary objective of SEROCoV-KIDS was to establish a population-based cohort of children and adolescents in the canton of Geneva rather than to estimate a single predefined parameter. Representativeness primarily depends on the sampling strategy and participation patterns rather than on sample size alone. To enhance representativeness, participants were randomly selected from population registries covering all children residing in the canton of Geneva.
The sample size was instead determined to ensure adequate statistical power for comparisons between key subgroups defined by serological status, age group, socioeconomic level, and the presence of chronic disease or disability. Subgroup sample sizes ranging from 100 to 1,000 participants were expected to provide at least 80% power to detect differences of 5–10% between groups, depending on the prevalence of the outcome. We have clarified this point in the manuscript.

2. Could you explain more on how random sampling was done from state registries?

We thank the reviewer for this question. We have clarified the sampling procedures in the manuscript by specifying that the random selection of eligible children and adolescents was performed by the Swiss Federal Office of Statistics or the Cantonal Office for Population and Migration using population registries covering residents of the canton of Geneva.

3. Please provide the rationale for recruiting Children with chronic health conditions who were referred by specialist physicians working at the Children's Hospital (Geneva University Hospitals): Were these the only source of children with chronic health conditions in Geneva?
The physician-referred clinical sample was not intended to be the sole source of children with chronic health conditions in the study. Children with chronic conditions could also be included through the population-based sample if they were randomly selected from the population registries. Recruitment through specialist physicians at the Geneva University Hospitals, the main pediatric referral center in the canton, was implemented to supplement the population-based sample and ensure adequate representation of children with a broad range of chronic health conditions. We have clarified this rationale in the manuscript.

4. Suggest clarifying if the participants recruited in the pilot phase were also included in the main study.
This is a good point. We have clarified in the manuscript that participants enrolled in the pilot phase were not included in the main cohort, unless they were also randomly selected for the main cohort. The pilot study was conducted solely to validate the study procedures before the launch of participant recruitment for the main study.

5. On the future plans for this cohort, suggest to consider how the cohort can be continued as in the context of future COVID-like pandemics (if possible & if funding can be secured).
We thank the reviewer for this valuable suggestion. We agree that one of the strengths of an established cohort infrastructure such as SEROCoV-KIDS is its potential to rapidly generate evidence in the context of future public health emergencies. We have therefore expanded the future plans section to acknowledge that, subject to securing additional funding, the cohort could provide a platform for monitoring the impact of future pandemics or other major societal crises on child and adolescent health and well-being.

VERSION 2 - REVIEW

**Reviewer**	**1**
**Name**	**Tung, Tao-Hsin**
**Affiliation**	**Taizhou Hospital of Zhejiang Province, Evidence-based Medicine Center**
**Date**	**14-Jul-2026**
**COI**	

I believe this manuscript could be accepted.

**Reviewer**	**2**

Name Chaw, Liling

Affiliation University of Brunei, PAPRSB Institute of Health Sciences

Date 03-Jul-2026

COI

The authors have fully addressed the comments raised by the reviewers.
